# Antibacterial, anti-HIV-1 protease and cytotoxic activities of aqueous ethanolic extracts from *Combretum adenogonium* Steud. Ex A. Rich (Combretaceae)

**DOI:** 10.1186/1472-6882-12-163

**Published:** 2012-09-26

**Authors:** Novatus F Mushi, Zakaria H Mbwambo, Ester Innocent, Supinya Tewtrakul

**Affiliations:** 1 Institute of Traditional Medicine, Muhimbili University of Health and Allied Sciences, Box 65001, Dar Es Salaam, Tanzania; 2 Department of Pharmacognosy and Pharmaceutical Botany, Faculty of Pharmaceutical Sciences, Prince of Songkla University, Hat Yai, Songkhla 90112, Thailand

**Keywords:** *Combretum adenogonium*, Combretaceae, Anti-HIV-1 protease, Antibacterial, Cytotoxicity

## Abstract

**Background:**

Records have shown that *Combretum adenogonium* Steud. Ex A. Rich (Combretaceae) is used in traditional medicine systems of several tribes in Tanzania. This study focused on the investigation of antibacterial activity, anti-HIV-1 protease activity, toxicity properties and classes of phytochemicals in extracts from *C. adenogonium* Steud. Ex A. Rich (Combretaceae) to evaluate potential of these extracts for development as herbal remedies.

**Methods:**

Dried plant material were ground to fine powder and extracted using 80% aqueous ethanol to afford root, leaf and stem bark extracts. The extracts were assayed for anti-HIV-1 protease activities, antibacterial activities using microdilution methods and cytotoxicity using brine shrimps lethality assay. Screening for major phytochemical classes was carried out using standard chemical tests.

**Results:**

All extracts exhibited antibacterial activity to at least one of the test bacteria with MIC-values ranging from 0.31-5.0 mg/ml. Two extracts, namely, root and stem bark exhibited anti-HIV-1 PR activity with IC_50_ values of 24.7 and 26.5 μg/ml, respectively. Stem bark and leaf extracts showed mild toxicity with LC_50_ values of 65.768 μg/ml and 76.965 μg/ml, respectively, whereas roots were relatively non-toxic (LC_50_ = 110.042 μg/ml). Phytochemical screening of the extracts indicated presence of flavonoids, terpenoids, alkaloids, tannins, glycosides and saponins.

**Conclusion:**

These results provide promising baseline information for the potential development of *C. adenogonium* extracts in treatment of bacterial and HIV/AIDS-related opportunistic infections.

## Background

The escalating number of HIV/AIDS cases in sub-Saharan African countries has dramatically raised high demand for medical care in health facilities throughout this resource-poor region. Treatment of HIV/AIDS is limited due to unavailability and high costs of antiretroviral drugs (ARVs) together with limited infrastructure for monitoring of HIV/AIDS patients. Phytomedicines have shown great promise in the treatment of infectious diseases including AIDS-related opportunistic infections [1]. Records indicate that the majority of traditional healers in Eastern, Southern and Western Africa use Combretaceae species for treatment of conditions like abdominal disorders, backache, bilharzia, cancer, coughs, colds, conjunctivitis, diarrhea, dysentery, dysmenorrhoea, fever, gastric ulcers, general weakness, venereal diseases, headaches, heart diseases, hypertension, jaundice, leprosy, nosebleeds, oedema, pneumonia, skin diseases, sore throats, dental caries, diabetes, enteralgia, eye diseases, general fatigue, hiccups, loss of appetite, malaria, menorrhagia, tuberculosis, tumours, HIV/AIDS infections, wasting and yellow fever [2-5]. Plants of the *Combretum* and *Terminalia* genera constitute majority of the Combretaceae family that are widely represented in Tanzania. At least 55 and 17 species of *Combretum* and *Terminalia*, respectively, are reported to be growing in Tanzania ranging from climbers, shrubs and big trees [6]. Most of these species are also found in other parts of tropical and warm temperate regions of the world [5,6]. *Combretum adenogonium* Steud. Ex A. Rich (Combretaceae) (syn:*Combretum fragrans* F. Hoffm or *Combretum ghasalense* Engl. & Diels) is a shrub or a small tree which grows up to 10-12 m high. It is common in deciduous woodland (Miombo) and wooded grassland associated with seasonally waterlogged clay soils and is sometimes also found on shallow, stony soils [6]. In various parts of Africa, the plant is used for treatment of leprosy, cough and syphilis, snakebite, aphrodisiac, diarrhea, new and chronic wounds, malaria and even septic wounds and fungal infection of the scalp [7,8]. Root, leaf and stem bark extracts of this plant have been investigated and established as having antifungal [9-11], antibacterial [11,12] and antiproliferative [8] properties. Stem bark of *C. adenogonium* have shown to exhibit significant *Clostridium chauvoei* neuraminidase enzyme inhibitory activity [13]. Previous phytochemical analyses have shown that, extracts of stem barks, root and leaves of *C. adenogonium* contain flavonoids, tannins and few saponins [10,14]. Furthermore, chemical analyses have shown that two phytosterols (β-sitosterol and stigmasterol) were isolated from the stem bark of *C. adenogonium*[15]*.*

The current study investigated antibacterial, anti HIV-1 protease and cytotoxic activities of extracts of *C. adenogonium* as part of our continued efforts to explore biological activities of plants of the Combretaceae for possible development of phytodrug to be used in managing HIV and AIDS-related opportunistic infections.

## Methods

### Materials

Ethanol (absolute) was purchased from Fluka Chemie GmbH (Sigma-Aldrich®, Zwijndrecht, Netherlands), Dimethyl sulfoxide (DMSO) was from Sigma® (Poole, Dorset, UK) whereas Tryptone Soya agar and broth from HIMEDIA® (Himedia Laboratories Pvt Ltd, Mumbai, INDIA). Iodonitrotetrazolium chloride was bought from SIGMA® (Sigma- Aldrich®, St Louis, USA). 96 well microtitre plates supplied by KAS medics (Tanzania). Recombinant HIV-1 PR, substrate peptides and acetyl pepstatin were purchased from Sigma Chemical Co., St Louis (USA). Standard bacterial strains that were used for screening extracts were *Escherichia coli* (ATCC 25922), *Pseudomonas aeruginosa* (ATCC 29953), *Salmonella typhi* (NCTC 8385), *Bacillus anthracis* (NCTC 10073), *Staphylococcus aureus* (NCTC 25923), *Klebsiella pneumoniae* (NCTC 9633), *Bacillus cereus* (NCTC 7494) and two clinical isolates *Streptococcus faecalis* (clinical isolate) and *Shigella flexneri* (clinical isolate). All strains were obtained from the Department of Microbiology, Muhimbili University of Health and Allied Sciences, Dar es Salaam, Tanzania.

The Brine Shrimps eggs were purchased from Aquaculture innovations (Grahamstown 6140, South Africa) and sea salt was prepared locally by evaporating water collected from the Indian Ocean, along the Dar es Salaam coast.

### Collection and extraction of plant materials

Roots, leaves and stem bark of *C. adenogonium* were collected from Handeni district, Tanga region, Tanzania. The plant was identified by Haji O. Seleman of the University of Dar es salaam, Botany Department. Herbarium specimen (voucher specimen collection number LBM 965) is deposited in the Herbarium of the Botany Department, University of Dar es Salaam. The collected plant material was air dried, pulverized and extracted with 80% aqueous ethanol at room temperature for 24hrs. The extracts were dried under *vacuo* followed by freeze-drying before analysis.

### Determination of antibacterial activity

Antibacterial activity of extracts was determined against 9 strains of bacteria (4 Gram positive and 5 Gram negative) and their minimum inhibitory concentrations (MICs) were assayed through two fold microdilution method using sterile 96-well microtitre plates [16]. Each well of the plates were first preloaded with 50 μl of the broth media followed by an addition of 50 μl of the extract (≤ 80mg/mL) into the first wells of each row tested. The resulting mixture were serially two fold diluted with tryptone soya broth media (made by dissolving 7.5g of tryptone soya broth in 250ml of sterilized distilled water) for each case 50 μl were drawn from the first row wells and transferred into the next and subsequent row wells. The remaining 50 μl from the last row well were discarded. Thereafter, 50 μl of the bacterial suspension (0.5 McFarland standard turbidity) was added in each well. Gentamycin sulphate was used as a positive control, DMSO as negative control while the rows with tryptone soya broth and bacteria only were used as growth controls. Both plates were incubated at 37°C for 24 h. MIC-values were determined by adding 20 μl of 0.02% p-iodonitrotetrazolium (INT) chloride dye in each well followed by incubation for 1 h at 37°C. The MIC-values of each extract was read at the concentration where a marked reduction in colour formation due to bacterial growth inhibition was noted.

### Anti-HIV-1 protease inhibitory activity test

This assay was carried out according to previous report [17]. In brief, the recombinant HIV-1 PR solution was diluted with a buffer composed of a solution containing 50mM of sodium acetate (pH 5.0), 1 mM ethylenediamine disodium (EDTA.2Na) and 2 mM 2-mercaptoethanol (2-ME) and mixed with glycerol in the ratio of 3:1. The substrate peptide, Arg-Val-Nle-(pNO2-Phe)-Glu-Ala-Nle-NH2, was diluted with a buffer solution of 50 mM sodium acetate (pH 5.0). 2μl of plant extract and 4 ml of HIV-1 PR solution (0.025 mg/ml) were added to a solution containing 2 ml of 50 mM buffer solution (pH 5.0) and 2 ml of substrate solution (2 mg/ml), and the reaction mixture (10 ml) was incubated at 37°C for 1 h. Acetyl pepstatin was used as a positive control and the reaction was performed under the same conditions. The reaction was stopped by heating the reaction mixture at 90^o^C for 1 min. Subsequently, 20 ml of sterilized water was added and an aliquot of 10 ml was analyzed by HPLC using RP-18 column (4.6 × 150 mm I.D., Supelco 516 C-18-DB 5 mm, USA). 10μl of the reaction mixture was injected to the column and gradiently eluted with acetonitrile (15-40%) and 0.2% trifluoroacetic acid (TFA) in water, at a flow rate of 1.0 ml/min. The elution profile was monitored at 280 nm. The retention times of the substrate and p-NO_2_-Phe-bearing hydrolysate were recorded at 11.25 and 9.72 min, respectively. The inhibitory activity on HIV-1 PR was calculated as follows:

$$\%\text{inhibition}=\left({\text{A}}_{\text{control}}-{\text{A}}_{\text{sample}}\right)\times 100/{\text{A}}_{\text{control}}$$

Whereas A is a relative peak area of the product hydrolysate [17].

### Brine shrimps lethality test

Brine shrimps lethality test (BST) was used to assay cytotoxicity activity of extracts [18]. Briefly, stock solutions (40 mg/ml) of each plant extracts was prepared by dissolving them in dimethyl sulfoxide (DMSO). Different levels of concentrations (240, 120, 80, 40, 24 and 8 μg/ml) were prepared by drawing different volumes from the stock solutions and then added into vials, each containing ten brine shrimps larvae. The volume was adjusted to 5 ml with artificial sea water prepared by dissolving 3.8 g of sea salt in 1 L of distilled water. Each level of concentration was tested in duplicate. The negative control contained brine shrimps, artificial sea water and DMSO (0.6%) only. The vials were then incubated under light for 24 h. The dead larvae were counted and mean was subjected to analysis using Fig P computer program (Biosoft Inc, USA).

### Phytochemical screening test

Chemical test was carried out on 80% aqueous ethanolic extracts to identify the constituents using standard method [19,20].

#### Test for tannins

About 1.0 g of the dried plant extract was boiled in 20 ml of water in a test tube and then filtered. A few drops of 0.1% ferric chloride was added and observed for brownish green or a blue-black colouration an indication of presence of tannins.

#### Test for saponins

About 2 g of the extract was boiled in 20 ml of distilled water in a water bath and filtered. 10 ml of the filtrate was mixed with 5 ml of distilled water and shaken vigorously for a stable persistent froth. The frothing was mixed with 3 drops of olive oil and shaken vigorously, then observed the formation of emulsion which indicates presence of saponins.

#### Test for flavonoids

Two methods were used to determine the presence of flavonoids in the plant extract. Dilute ammonia solution (5ml) were added to a portion of the aqueous filtrate of each extract followed by addition of concentrated H_2_SO_4_. A yellow colouration observed in each extract indicated the presence of flavonoids. The yellow colouration disappeared on standing.

A portion of the extract was in each case heated with 10 ml of ethyl acetate over a steam bath for 3 min. The mixture was filtered and 4 ml of the filtrate was shaken with 1 ml of dilute ammonia solution. A yellow colouration was observed indicating a positive test for flavonoids.

#### Test for terpenoids

5 ml of each extract was mixed in 2 ml of chloroform, and carefully 3 ml of concentrated H_2_SO_4_ was added to form a layer. A reddish brown colouration of the inter face was formed to show positive results for the presence of terpenoids.

#### Test for glycosides

0.5 gm of each sample was stirred with 10 ml of boiling distilled water. This was filtered and 2 ml of the filtrate hydrolyzed with few drops of concentrated HCl and the solution rendered alkaline with a few drops of ammonia solution. About 5 drops of this solution was added to 2 ml of benedict’s quantitative reagent and boiled. Appearance of reddish brown precipitate showed presence of glycosides.

### Data analysis

The results of anti-HIV-1 PR activity were expressed as means ± SD of three determinations. The IC_50_ values were calculated using the Microsoft Excel programme with values greater than 100 μg/ml referred to as inactive for extracts [17]. The mean results of brine shrimp mortality against the logarithms of concentrations were plotted using the Fig P computer program (Biosoft Inc, USA), which also gives regression equations. Regression equations were used to calculate LC_16_, LC_50_ and LC_84_ values. Confidence intervals (95% CI) were calculated according to a previously reported method [21]. An LC_50_ value greater than 100 μg/ml was considered to represent an inactive extract [22]. The MIC-values are interpreted as follows; 0.05-0.5 mg/ml - strong activity, 0.6-1.5 mg/ml - moderate activity and above 1.5 mg/ml - weak activity [23].

## Results

### Antibacterial activity

The antibacterial activity of aqueous ethanolic extracts from the root, stem bark and leaf of *C. adenogonium* are presented in Table 1. All plant extracts showed activities to at least one of the test bacteria with MIC-values ranging from 0.31-5.0 mg/ml except for *S. flexneri* which was less susceptible (Table 1). Moderate activity for stem bark extract was observed for *B. cereus, P. aeruginosa* and *S. typhi* each with MIC-value of 1.25 mg/ml. The root extract exhibited antibacterial activity against all the test organisms. Leaf extract was the most active against Gram-positive organisms namely *B. cereus*, *B. anthracis* and *S. faecalis* with MIC-values of 0.31, 0.31 and 0.63 mg/ml, respectively (Table 1).

**Table 1:** **Antibacterial activity of aqueous ethanolic extracts of*C. adenogonium***

**Test organisms**	**Minimum Inhibition Concentration (MIC) in mg/ml**			
	**Stem bark extract**	**Root extract**	**Leaf extract**	**+ve (gentamycin)**
*Kb. Pneumonia*	5.0	5.0	5.0	0.0025
*E. coli*	5.0	5.0	1.25	0.0016
*B. cereus*	1.25	1.25	0.31	0.0013
*P. aeruginosa*	1.25	1.25	1.25	0.0016
*S. aureus*	2.5	2.5	5.0	0.00078
*S. typhi*	1.25	1.25	1.25	0.0063
*S. flexneri*	>10.0	1.25	>10.0	0.0025
*B. anthracis*	2.5	2.5	0.31	0.0025
*S. faecalis*	>10.0	2.5	0.63	0.000024

### Anti-HIV-1 protease activity

Table 2 shows results of the activity of root, stem bark and leaf extracts of *C. adenogonium* against HIV-1 protease inhibition activities. Root and stem bark extracts exhibited moderate activity with IC_50_ values of 24.7 and 26.5 μg/ml. Acetyl pepstatin was used as a positive control with an IC_50_ value of 2.2 μg/ml. Leaf extract was found to be inactive (IC_50_ > 100 μg/ml).

**Table 2:** **Anti-HIV-1 protease activity of aqueous ethanolic extracts of *C. adenogonium***

**Sample name**	**% Inhibition at various concentrations (μg/ml)**			
	**10**	**30**	**100**	**IC**_**50**_**(μg/ml)**
Stem bark extract	27.6 ± 1.4	60.4 ± 1.4	70.9 ± 4.2	26.5
Leaf extract	-	-	17.4 ± 1.4	>100
Root extract	21.4 ± 0.9	66.7 ± 1.1	79.0 ± 1.8	24.7
**Acetyl** pepstatin*	70.0 ± 0.5	82.4 ± 0.4	88.5 ± 0.2	2.2

**Key:** The results are Mean ± SD (n = 3), * positive control, - no inhibition.

### Brine shrimps test results

Results of the activity of stem bark, leaf and root extracts against brine shrimp larvae are shown in Table 3. Leaf and stem bark extracts possessed mild toxicity to brine shrimps with LC_50_ values of 65.768 and 76.965 μg/ml, respectively. Leaf extract was non-toxic (LC_50_ = 110.042 μg/ml).

**Table 3:** **Brine shrimp toxicity of aqueous ethanolic extract of *C. adenogonium***

**Plant plant**	**LC**_**50**_**(μg/ml)**	**95%Confidence interval (CI)**	**Regression equation**	**Regression coefficient (r**^**2**^**)**
Root extract	110.042	86.989 – 139.203	Y = 92.283logx – 138.460	0.926
Leaf extract	76.965	63.660 – 93.051	Y = 113.890logx – 164.830	0.927
Stem bark extract	65.768	53.688 – 80.566	Y = 106.710logx – 144.410	0.943
Cyclophosphamide*	16.37	12.01 – 22.31	Y = 69.968logx – 34.936	0.995

**Key:** * positive control.

### Phytochemical screening results

Results on preliminary phytochemical screening are shown in Table 4. All extracts tested positive for presence of tannins. Flavonoids and saponins were detected to be present in both the root and leaf extracts whereas alkaloids were detected in stem bark only. While terpenoids were detected only in the leaf extract, glycosides were detected in root and stem bark extracts.

**Table 4:** **Phytochemical screening of the extracts from *C. adenogonium***

**Extract origin**	**Classes of compounds tested**					
	**Taninns**	**Alkaloids**	**Flavonoids**	**Terpenoids**	**Glycosides**	**Saponins**
Root	+	-	+	-	+	+
Leaf	+	-	+	+	-	+
Stem bark	+	+	-	-	+	-

**Key:** +: present; -: absent.

## Discussion

The antibacterial activity of aqueous ethanolic extracts from the root, stem bark and leaf of *C. adenogonium* was determined by screening extracts against standard bacterial strains and clinical isolates. All plant extracts showed activities to at least one of the test organism. Leaf extract was the most active against *B. cereus*, *B. anthracis* and *S. faecalis* with MIC-values of 0.31, 0.31 and 0.63 mg/ml, respectively, but had no activity against *S. flexneri*. The root extracts displayed antibacterial activity against all the test organisms. Similarly, stem bark extracts showed activity as the root extracts except for *S. flexneri* and *S. faecalis* in which there was no activity observed. These results corroborate well with previous investigations where records indicate that methanolic extracts of the root and the leaf of *C*. *adenogonium* showed activity against *S*. *aureus*[12]. In the present study however, all the extracts showed activity against *E.coli*, contrary to what was previously reported that both the leaf and root extracts had no activity against *E. coli*[12]. The differences however, might be contributed by the type of solvent that was used for extraction of plant components. In the present study, 80% aqueous ethanol was used as a solvent which might have extracted more polar constituents compared to methanolic extracts, which were used in the previous investigation [12]. Antimicrobial results from the present and previous investigations [11,12], lends support to further *in vivo* and other trials to validate the use of *C. adenogonium* in traditional medicine systems for treatment of diseases like diarrhea [12], coughs, syphilis [24], fresh wounds [25] and septic wounds [7].

Aqueous ethanolic extracts of root and stem bark have exhibited moderate anti-HIV-1 protease inhibitory activity with IC_50_ value of 24.7 and 26.5 μg/ml, respectively, compared to Acetyl pepstatin which was used as a positive control with an IC_50_ value of 2.2 μg/ml (Table 2). Leaf extract which was found to be inactive in this study, was previously reported to have anti-HIV-1 activity with an IC_50_ value of 2.7μg/ml [26]. Thus, both root, leaf and stem bark extracts of *C. adenogonium* have anti-HIV properties, although mechanism of action or composition may be different. The anti-HIV-1 activity of *C. adenogonium* and its use in managing HIV/AIDS diseases is well supported by other species of Combretaceae [26-28]. The present study also showed that stem bark and leaf extracts exhibited some toxicity against brine shrimps while roots were virtually non-toxic (Table 3). Our findings portrayed similar observations as previously reported for *in vitro* antiproliferative activity [8] hence indicating this plant’s potential of being antiseptic and antitumor agent.

The present phytochemical screening results revealed presence of major classes of constituents including tannins, alkaloids, flavonoids, terpenoids, glycosides and saponins as presented in Table 4. These classes of compounds are reported to exhibit a variety of biological activities including antiviral, antibacterial, anti-inflammatory and analgesic all of which are relevant to the traditional uses of this plant as claimed [29-31]. Presence of glycosides which were detected in the root and stem bark extracts only could be speculated as the major contributing factor for the anti-HIV-1 protease inhibitory activity in these extracts compared to other constituents for example, saponins and flavonoids that were detected in both the roots and leaves and tannins that were found in all the three extracts.

## Conclusion

The antibacterial, anti-HIV-1 protease and cytotoxic activities exhibited by aqueous ethanolic extracts of *C. adenogonium*, lends support to further *in vivo* and other trials to validate the potential of this plant in the development of phytodrug for treatment of various ailments including HIV/AIDS-related opportunistic infections.

## Competing interests

The authors declare that they have no competing interests.

## Authors’ contributions

NFM conceptualized the study, developed study protocol, contributed to data acquisition and analysis, and preparation of the manuscript. ZHM supervised the study and preparation of the manuscript. EI developed study protocol, contributed to data analysis and preparation of the manuscript. ST performed the HIV-1 protease assay and analysis of data. All authors read and approved the final manuscript.

## Pre-publication history

The pre-publication history for this paper can be accessed here:

http://www.biomedcentral.com/1472-6882/12/163/prepub
